# Supplementary material for: In Vitro Antifungal Activity of Selected Essential Oils against Drug-Resistant Clinical Aspergillus spp. Strains
Source: Molecules. 2023 Oct 25;28(21):7259. doi: 10.3390/molecules28217259 (PMC10650698; doi:10.3390/molecules28217259)
Supplement: Supplementary file 1 [file molecules-28-07259-s001.zip › molecules-2629026-supplementary.pdf]

Table S1 Essential oils used and their major components

Essential oil										
Clove	Eucalyptus	Geranium	Hybrid lavender	Lavender	Lemon	Lemongrass	Neroli	Oregano	Thyme red	Tea tree
Major components										
78.91% Eugenol	80.64% 1-8 Cineole	33.22% Citronellal + Neral	33.83% Linalol	27.11% Linalol	69.25% Limonene	22.64% Geraniol	31.49% Linalol	62.61% Carvacrol	26.5% Thymol	35.88% Terpinen-4-ol
11.64% Eugenyl acetate	10.41% Limonene	15.56% Geraniol-formate	27.43 % Linalyl acetate	24.4% Linalyl acetate	11.37% β-pinene	7.74% Limonene	17.32% β-Pinene	12.36% ρ-cymene	16.2% ρ-Cymene	19.65% γ-Terpinen
6.04% β-Caryophyllene	3.65% ρ-Cymene	5.71% iso-menthone	7.10% Camphor	9.78% β-Ocimene	7.86% γ-Terpinen	7.66% Canphene	15.76% Limonene	3-9% γ-Terpinen	13.2% Limonene	8.64% α-Terpinen
0.69% α-Humulene	2.52% α-Pinene	4.19% Linalol	4.84% 1,8 Cineole	5.36% β-Caryophyllene	1.98% Sabinene	6.81% Methyl isoeugenol	6.67% β-Ocimene	0.5-5% Thymol	11.5% α-Pinene	4.61% ρ-cymene
0.27% α-Copaene	0.49% α-Phellandral		4.18% Borneol	5.11% 4-Terpineol	1.75% α-Pinene	5.90% Geranyl acetate	4.04% Linalyl acetate + geraniol	0.5-4% β-Caryophyllene	7.8% Carvacrol	4.07% 1,8-Cineole
									0.1-0.5% Eugenol	
